# Loss of Dgcr8-mediated microRNA expression in the kidney results in hydronephrosis and renal malformation

**DOI:** 10.1186/s12882-015-0053-1

**Published:** 2015-04-14

**Authors:** Malte P Bartram, Claudia Dafinger, Sandra Habbig, Thomas Benzing, Bernhard Schermer, Roman-Ulrich Müller

**Affiliations:** Department II of Internal Medicine and Center for Molecular Medicine, University of Cologne, Kerpener Str. 62, Cologne, 50937 Germany; Department of Pediatrics, University of Cologne, Cologne, Germany; Cologne Excellence Cluster on Cellular Stress Responses in Aging-Associated Diseases, University of Cologne, Cologne, Germany; Systems Biology of Ageing Cologne, University of Cologne, Cologne, Germany

**Keywords:** CAKUT, Dgcr8, Dicer, Hydronephrosis, Kidney, miRNA

## Abstract

**Background:**

Small non-coding RNA molecules (miRNAs) play a pivotal role in regulating gene expression in development. miRNAs regulate key processes at the cellular level and thereby influence organismal and tissue development including kidney morphogenesis. A miRNA molecule is initially synthesized as a longer hairneedle-shaped RNA transcript and then processed through an enzymatic complex that contains the RNA-processing enzyme Drosha and its essential interactor Dgcr8. Resulting pre-miRNAs are then cleaved by Dicer. Recent data showed that loss of *Dicer* resulted in severe developmental kidney phenotypes. However, as Dicer has multiple miRNA-independent functions, it was not entirely clear whether the observed renal phenotypes could be exclusively attributed to a lack of miRNA expression.

**Methods:**

We analyzed the role of miRNAs in kidney development by conditional gene deletion of *Dgcr8* in the developing kidney using a transgenic mouse line that expresses Cre recombinase in the distal nephron and derivatives of the ureteric bud in kidney development.

**Results:**

Animals with a gene deletion of *Dgcr8* in these tissues developed severe hydronephrosis, kidney cysts, progressive renal failure and premature death within the first two months after birth, a phenotype strongly resembling *Dicer* deletion.

**Conclusions:**

Here we show that conditional gene deletion of the essential miRNA-processing enzyme *Dgcr8* in the developing renal tubular system results in severe developmental defects and kidney failure. These data confirm earlier findings obtained in *Dicer* knock-out animals and clearly illustrate the essential role of miRNAs in kidney development. The data suggests that miRNA dysregulation may play an important, yet ill-defined role in the pathogenesis of inborn defects of the genitourinary system and indicate that miRNA defects may be causative in the development of human disease.

**Electronic supplementary material:**

The online version of this article (doi:10.1186/s12882-015-0053-1) contains supplementary material, which is available to authorized users.

## Background

MicroRNAs are important regulators of gene expression and have been shown to be crucial to developmental processes in many different tissues [1]. To study the role of miRNAs in the kidney several publications have addressed this question using a conditional knockout of *Dicer*, the RNAse III enzyme catalyzing the maturation from pre-miRNA to mature microRNAs [2-9]. This strategy revealed major defects in both tubular and glomerular development and maintenance. The loss of Dicer in derivatives of the ureteric bud and in the tubular system lead to a severe hydronephrosis coupled with cystic kidneys and loss of functional parenchyme [2-4,6]. These phenotypes strongly resemble congenital anomalies of the kidney and urinary tract (CAKUT) in the clinical setting. However, whether this is truly due to loss of microRNAs has remained elusive since Dicer fulfills several other important functions which may well be involved in renal development [10]. Among these are its role as a DNAse in genomic DNA fragmentation during apoptosis, the processing of endogenous siRNA and the detoxification of repeat elements [11-13]. In an elegant study elucidating the role of microRNAs in skin development this issue has been addressed using the conditional knockout of genes involved in different steps of miRNA processing displaying a phenotypic overlap [14,15]. As to the kidney this has been done successfully regarding the effects of podocyte-specific loss of microRNAs using a conditional knockout allele of *Drosha* [16], which confirmed previous studies based on *Dicer* knockout in podocytes [7-9].

Consequently, we set out to confirm the role of microRNAs in renal development using a conditional allele of *Dgcr8*. Dgcr8 interacts with Drosha and is essential for its role in processing pri-microRNAs in the nucleus [17].

## Methods

### Mice

*Dgcr8 fl/fl* animals were described before [18] and generously provided by Elaine Fuchs (Rockefeller University, NYC, USA). To generate a kidney tubulus specific *Dgcr8* knockout these mice were crossed to a *KspCre* transgenic line (contributed by Peter Igarashi, UT Southwestern Medical Center, Dallas, USA) that expresses the Cre recombinase under the control of a ksp-cadherin promotor resulting in Cre expression in the developing genitourinary tract and kidney tubulus system was performed as described before [19]. Animals were housed in standardized specific pathogen-free conditions in the animal facility of the CMMC (University of Cologne).

All animal procedures were performed according to European (EU directive 86/609/EEC), national (TierSchG), and institutional guidelines and were approved by local governmental authorities (LANUV NRW).

### Histology

The kidneys were fixed in formalin, embedded in paraffin and stained with PAS according to standard protocols. To analyse the expression of Ki-67, slides of fixed and paraffin-embedded mouse kidneys were de-paraffinized using Xylol and descending concentrations of ethanol. Antigen retrieval was carried out by warming kidney slides in citrate buffer (10 mM, pH6) for 10 min using a microwave. After blocking with 3% H_2_O_2_ and Avidin and Biotin (Vector Laboratories, Inc.) for 15 min each, slides were sequentially incubated with the Ki-67 antibody (rabbit Ki-67 ab16667, abcam, 1:500 dilution, over night at 4°C) and after washing with PBS with biotinylated anti-rabbit IgG (Jackson ImmunoResearch, West Grove, PA, USA; 1 h at room temperature). Kidney slides were labelled with ABC kit (Vector Laboratories, Inc.), and development was carried out using diaminobenzidine solution (Sigma Aldrich). Slides were counterstained with hematoxylin (Sigma-Aldrich), dehydrated and afterwards mounted with Histomount (National Diagnostics). Stained slides were scanned using a Slidescanner (Leica) and analyzed using the ImageScope software (version 12.0.1.5030, Aperio).

### Laboratory medicine

Heparinized blood was obtained by cardiac puncture. Plasma was prepared by centrifugation at 3000 rpm for 10 min. Urea was measured in the central laboratory medicine unit of the University Hospital of Cologne using the kinetic UV test (Roche Diagnostics). Significance was calculated using a two-tailed Student’s *t* test for all measurements (urea, body weight of mice).

### qPCR

RNA was extracted from whole mouse kidneys using acid guanidinium thiocyanate-phenol-chloroform extraction [20]. RT reactions were performed using the Taqman microRNA Reverse Transcription Kit (ABI). Expression of mir-192 (assay ID 000491), and miR-200b (assay ID 4426961) was analyzed using Taqman assays (ABI), and snoRNA135 (assay ID 001230) served as endogenous control. All qPCR experiments were performed on the ABI 7900HT System. All data points were generated using the number of biological replicates indicated in the figure. Data analysis and statistics were performed using the Expression Suite v1.3 software package applying the comparative Ct method and using one standard deviation for error bar calculation (LifeTechnologies).

### TUNEL assay

To analyse apoptosis in the kidneys of *Dgcr8* knockout and littermate control mice we utilized the Promega DeadEnd Fluorometric Kit according to the manufacturers protocol. Pictures were taken with an inverted microscope (Axiovert200, equipped with an ApoTome system and an AxioCam MRm camera. Objective used: Plan Apochromat 20×/0.8 NA. Carl Zeiss) using Axiovision 4.8 (Carl Zeiss).

## Results

To analyze the role of Dgcr8 and thereby miRNAs in the developing tubular system independent of a Dicer mouse model, we crossed a *Dgcr8 fl/fl* mouse line [18] with *KspCre* mice [19], resulting in a conditional knockout of *Dgcr8* in the developing urogenital tract and tubulus system.

**Figure 1:**
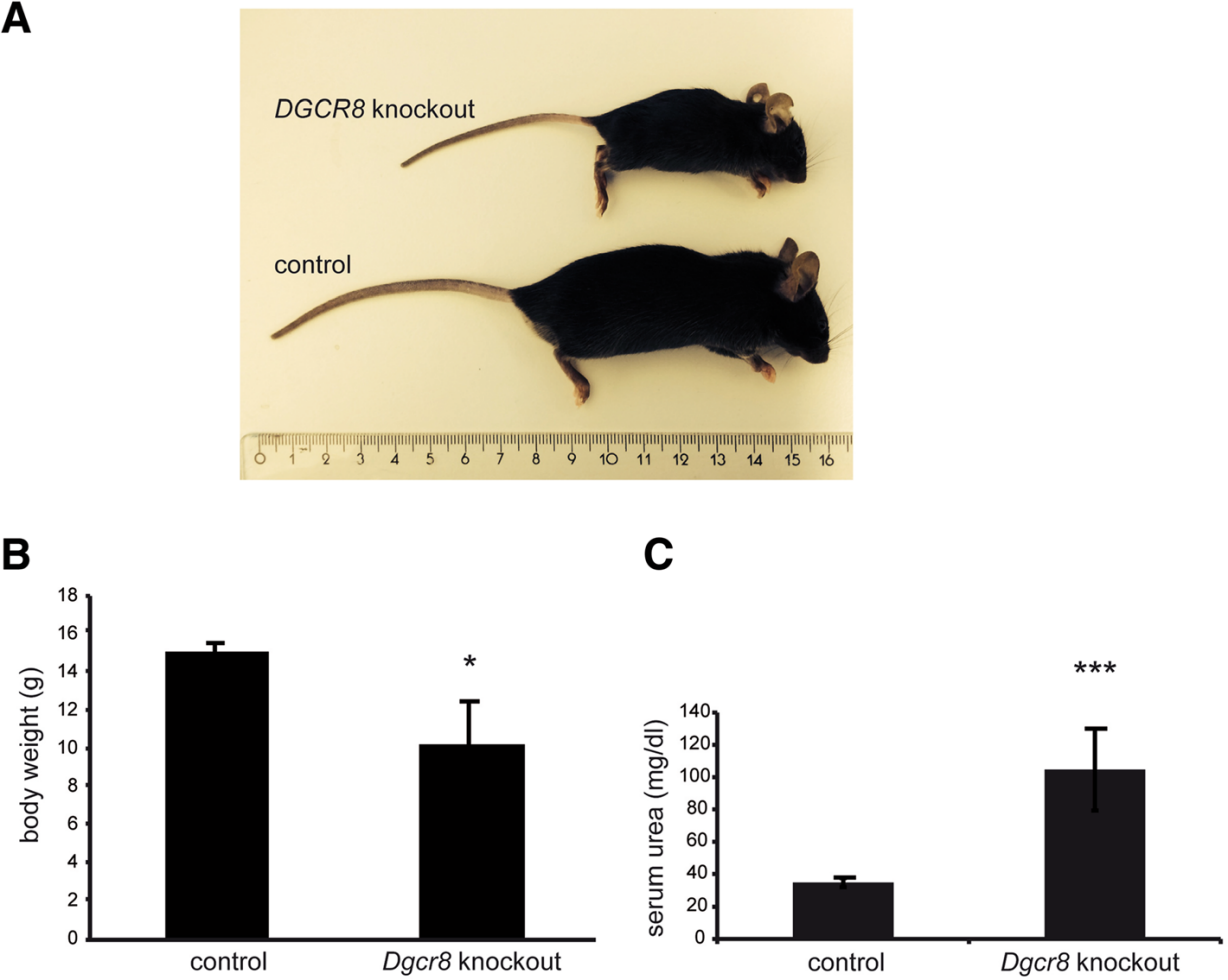
Kidney-specific knockout of *Dgcr8* results in end stage renal disease. **A** Conditional *Dgcr8 fl/fl; KspCre* knockout mice are markedly smaller than their control littermates (7 week old animals) **B** *Dgcr8* knockout mice have a significant lower body weight in comparison to their control littermates (4 week old animals; * = p < 0.05; error bars represent SEM; knockout: n = 3; control: n = 5) **C** *Dgcr8* knockout mice develop end stage renal disease as displayed by elevated serum urea levels in comparison to control animals (4–7 week old animals; *** = p < 0.001; error bars represent SEM; knockout: n = 6; control: n = 13).

**Figure 2:**
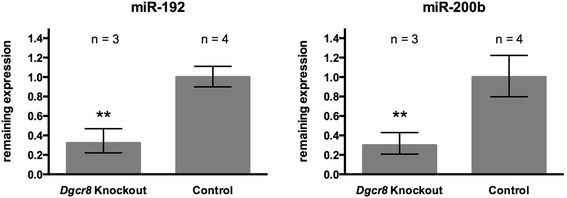
*KspCre*-mediated loss of *Dgcr8* induces depletion of tubulus-specific miRNAs. Both expression of miR-192 and miR-200b is strongly reduced in *Dgcr8* knockout kidneys when compared to WT littermates (4-7 week old animals; ** = p < 0.01; error bars represent SEM).

*Dgcr8 fl/fl; KspCre* positive mice (afterwards named *Dgcr8* knockout) showed an obvious delay in growth (Figure 1A) in comparison to the control littermates and a significantly reduced body weight (Figure 1B). This phenotype was most likely caused by the developing renal failure in these mice, since they showed a marked elevation in serum urea (Figure 1C). The phenotype became apparent in the first weeks of life, several mice died during the weaning period leading to a significantly reduced number of *Dgcr8* knockout animals after weaning in comparison to the control genotypes (Additional file 1: Figure S1A). None of the *Dgcr8* knockout mice analyzed so far survived longer than 8 weeks, most likely due to development of end stage renal disease. In order to confirm that loss of *Dgcr8* abrogates miRNA biogenesis we quantified two miRNAs that had been shown before to be primarily expressed in renal tubular cells and to be depleted by *KspCre* driven loss of *Dicer* [3,6,21]. Both expression of miR-192 and miR-200b were greatly reduced in the conditional *Dgcr8* knockout mouse line (Figure 2).

**Figure 3:**
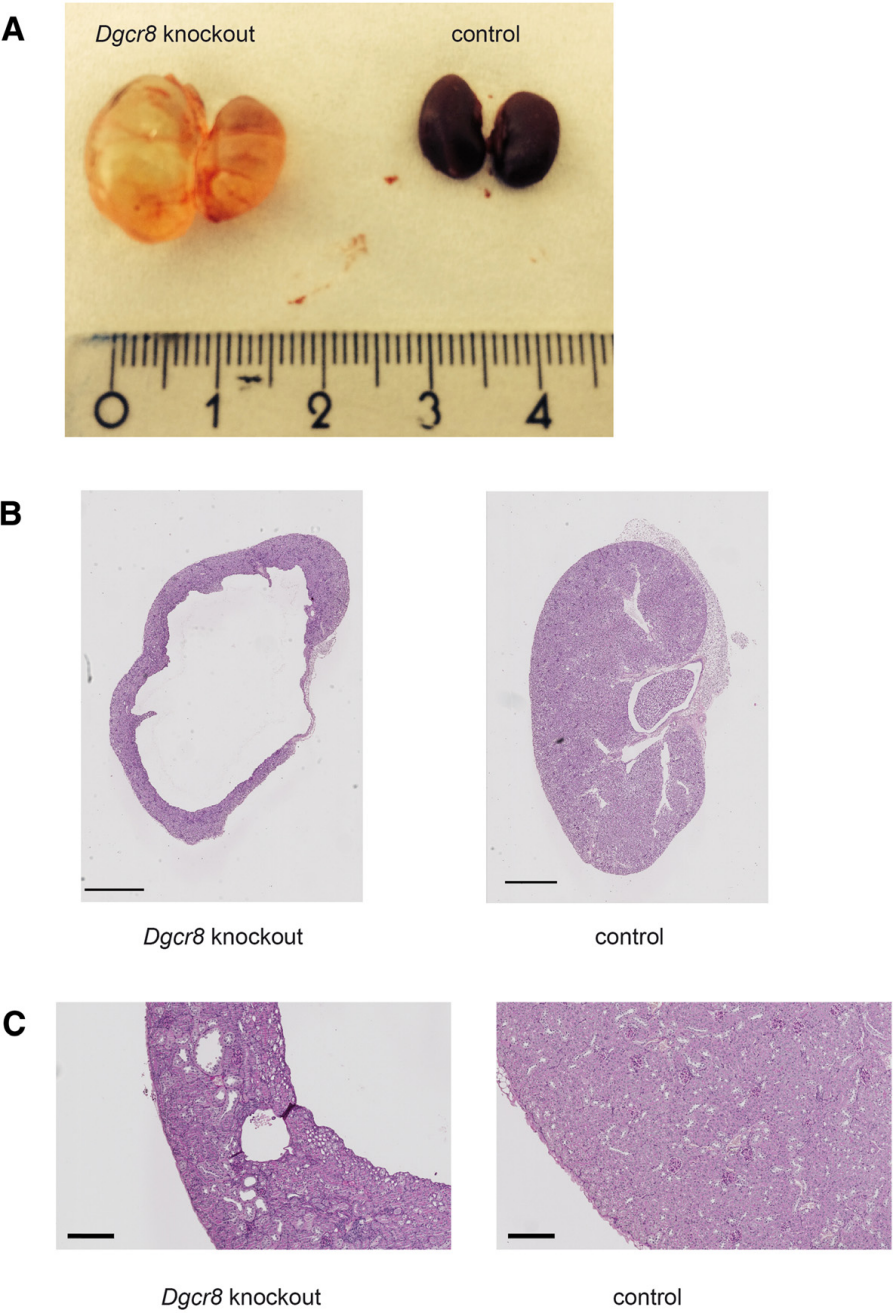
Hydronephrosis and cystic kidneys of *Dgcr8* knockout animals. **A** Conditional knockout of *Dgcr8* in the renal tubulus system leads to hydronephrosis. **B + C** Histological analysis confirms hydronephrosis with severe loss of kidney parenchyma especially in the medulla region, a thinned cortex and kidney cysts (bar = 1000 μm (**B**) and 200 μm (**C**)).

Further examination of the kidneys of the *Dgcr8* knockout mice showed the macroscopic picture of severe hydronephrosis with a dilated ureter and kidney pelvis (Figure 3A). There were no signs for a complete obstruction of the ureter, since the bladder of the knockout animals was filled with urine (data not shown).

**Figure 4:**
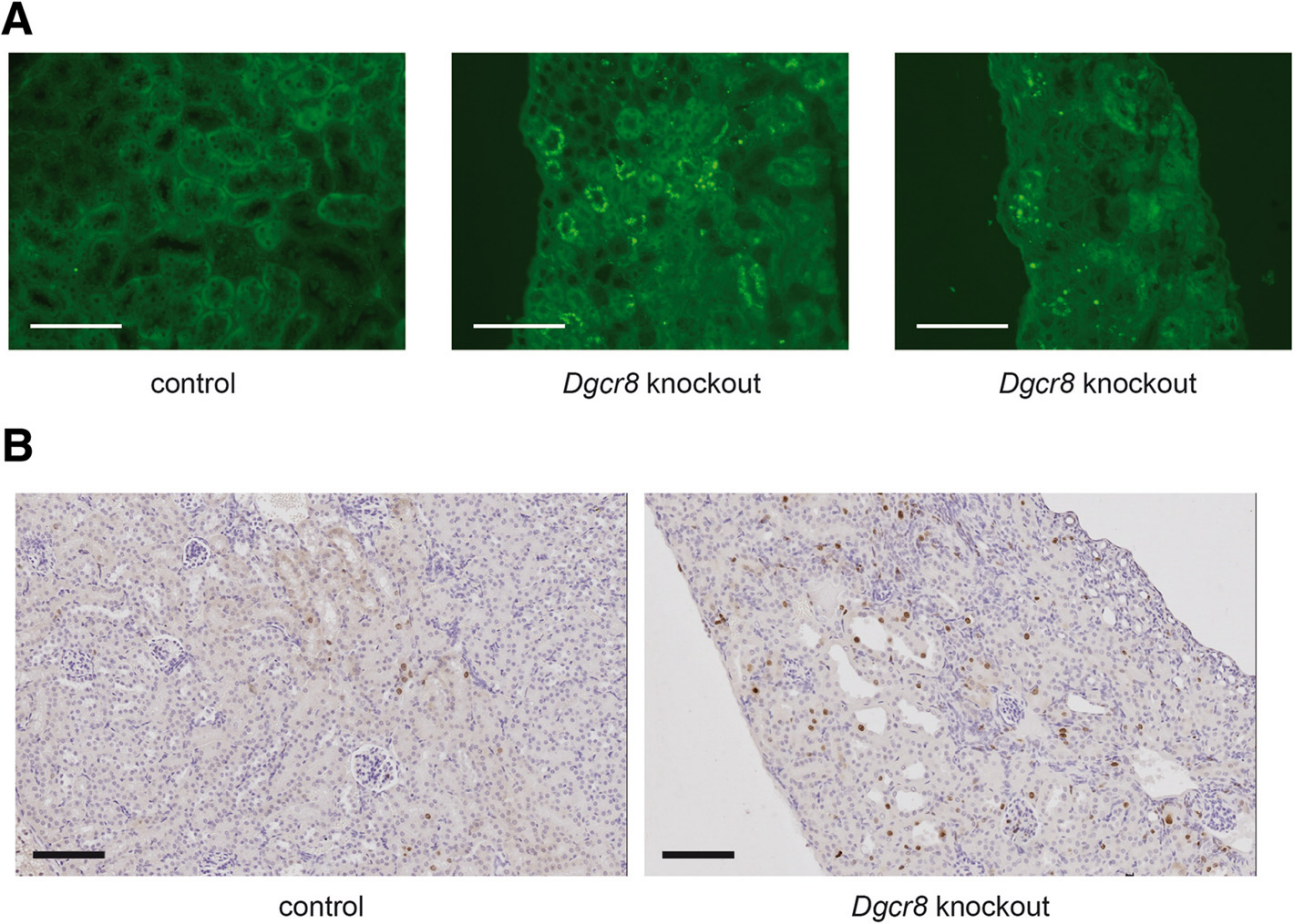
Loss of *Dgcr8* induces apoptosis and proliferation. **A** TUNEL staining reveals a dramatic increase in apoptosis in *Dgcr8* deficient kidneys (representative images of renal cortex, bar = 100 μm). **B** Staining for Ki-67 reveals an increase in proliferation in *Dgcr8* knockout kidneys (bar = 100 μm).

Histopathological analyses of the kidneys confirmed the diagnosis of hydronephrosis and obstructive nephropathy. In most affected animals nearly the entire medulla was missing, and the cortex was very thin (Figure 3B). In addition, some kidneys displayed dilated tubuli and cysts were observed (Figure 3C). As described for *Dicer* knockout mice before [3] the *Dgcr8* knockout kidneys showed a reduced glomerular density pointing towards a branching defect (Additional file 1: Figure 1B). To further analyse the cellular basis to this phenotype we performed TUNEL assays and Ki-67 stainings. These revealed a strong induction of apoptosis and cellular proliferation in tubular cells of *Dgcr8* knockout animals (Figure 4).

## Discussion

The loss of *Dgcr8* specifically in renal epithelial cells nicely resembled the phenotype observed earlier when knocking out *Dicer* in the same tissue using the same cre mouse line. Both Dicer and Dgcr8 are involved in a number of different processes primarily regarding the processing of nucleic acids [10-13,22,23]. Since the known overlapping function of these two genes is microRNA processing our study strongly supports the conclusion that the severe phenotype in both mouse models including hydronephrosis and renal failure is caused by the loss of microRNA processing. This finding is of great importance and encouraging to plan and perform follow-up studies now addressing the role of specific microRNAs in development and maintenance of renal architecture.

Interestingly, a small number of microRNAs is either independent from Dicer or from the Drosha/Dgcr8 complex [24,25]. As an example the so-called mirtrons are processed by the spliceosome in the nucleus instead of Drosha/Dgcr8 [26]. As for Dicer miR-451 is not processed by this enzyme but depends on Ago2 in its maturation [27-29]. Consequently, our study does not only confirm the crucial role of microRNAs in renal development but also narrows down the list by excluding any small RNAs processed by only one of the two enzymes.

In contrast to our previous study on *Dicer* knockout animals with a penetrance of the phenotype of about 66% [3] the *Dgcr8* knockout described in this study has a complete penetrance with no *Dgcr8* knockout animal surviving longer than 8 weeks. Whether this may be due to a partial rescue of the *Dicer* knockout animals by another enzyme – e.g. for microRNAs that are generally processed by Dicer but may be processed by Ago2 as well – remains elusive and will be subject to future studies.

In summary, our results underline the relevance of microRNAs during kidney development and will encourage further functional studies examining single microRNAs and their target mRNA interactions – such as miR-20 and its targets PKD1 and PKD2 [3,6,30,31] - as regulators of renal organogenesis. This will be fundamental for gaining a better understanding of developmental defects in human kidney formation as observed in CAKUT – the predominant cause of end-stage renal disease in children.

## Conclusions

MiRNAs are key regulators of intracellular signaling and development. In this study we show that loss of Dgcr8 dependent miRNAs in the kidney epithelium leads to severe hydronephrosis, kidney cysts and rapid kidney failure. This confirms an essential role for miRNAs in renal development and disease.

## Additional file

Additional file 1: Figure S1. A Genotyping after weaning at 3–4 weeks of age reveals that the knockout mice did not reach weaning at a Mendelian ratio suggesting death before the timepoint of weaning and genotyping. In line with this finding several mice of unknown genotype had died before weaning. B Glomerular density is reduced in *Dgcr8* knockout kidneys (n = 3 per group; error bars represent SEM; ** = p <0.01 using an unpaired Student’s *t*-test; 5 high power fields of the kidney cortex were counted per animal) C Table showing the number of mice revealing either kidney cysts or hydronephrosis at weaning.

## Abbreviations

CAKUT: Congenital anomalies of the kidney and urinary tract
Dgcr8: DiGeorge syndrome critical region 8
PKD: Polycystic kidney disease
