# Supplementary figures and images for: Loss of Dgcr8-mediated microRNA expression in the kidney results in hydronephrosis and renal malformation
Source: BMC Nephrol. 2015 Apr 14;16:55. doi: 10.1186/s12882-015-0053-1 (PMC4445526; doi:10.1186/s12882-015-0053-1)

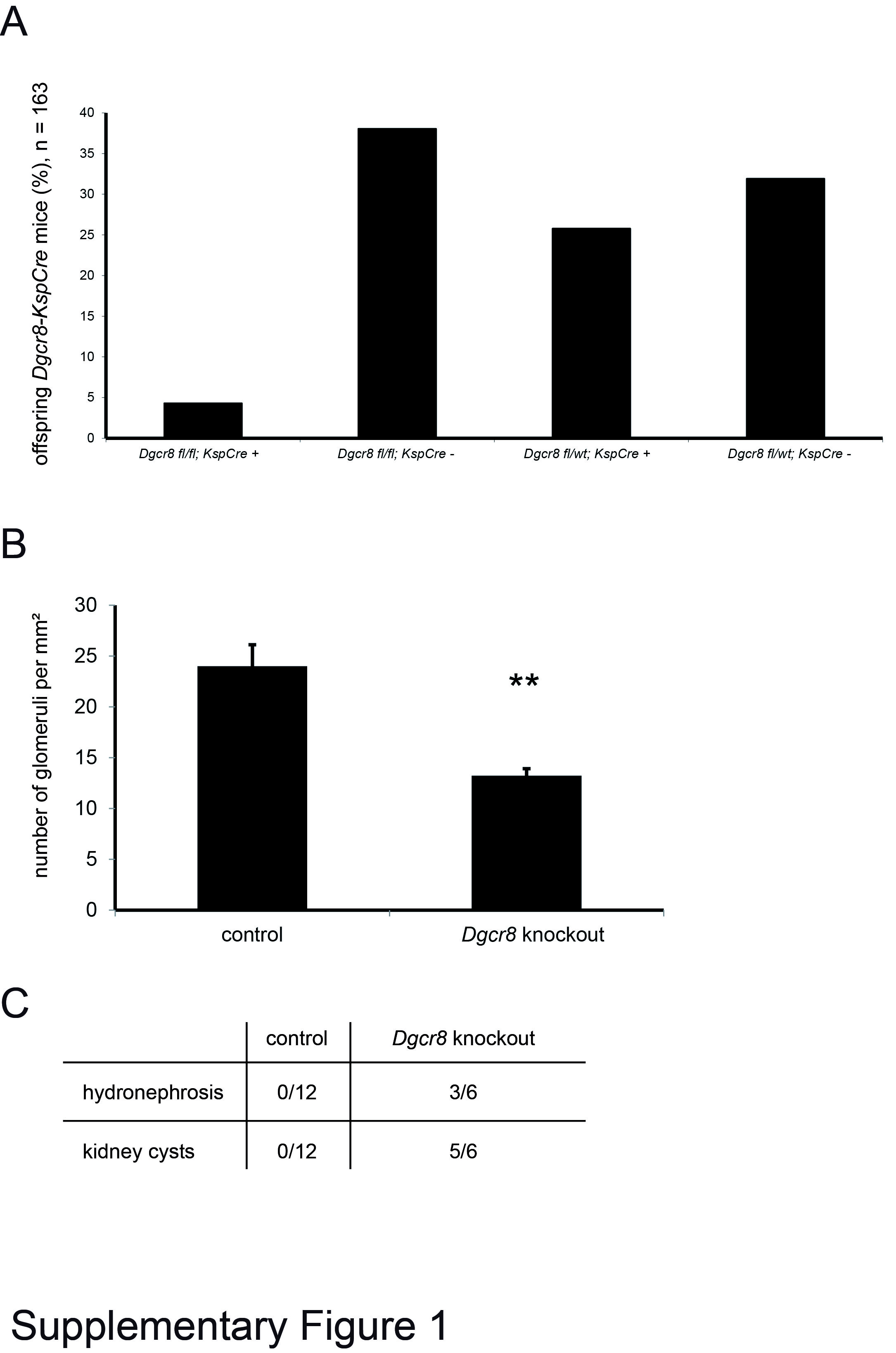

Supplement: Additional file 1: Figure S1. — A Genotyping after weaning at 3–4 weeks of age reveals that the knockout mice did not reach weaning at a Mendelian ratio suggesting death before the timepoint of weaning and genotyping. In line with this finding several mice of unknown genotype had died before weaning. B Glomerular density is reduced in Dgcr8 knockout kidneys (n = 3 per group; error bars represent SEM; ** = p <0.01 using an unpaired Student’s t-test; 5 high power fields of the kidney cortex were counted per animal) C Table showing the number of mice revealing either kidney cysts or hydronephrosis at weaning. [file 12882_2015_53_MOESM1_ESM.jpeg]
